# Subjective COVID-19-related work factors predict stress, burnout, and depression among healthcare workers during the COVID-19 pandemic but not objective factors

**DOI:** 10.1371/journal.pone.0270156

**Published:** 2022-08-12

**Authors:** András Spányik, Dávid Simon, Adrien Rigó, Mark D. Griffiths, Zsolt Demetrovics

**Affiliations:** 1 Doctoral School of Psychology, ELTE Eötvös Loránd University, Budapest, Hungary; 2 Institute of Psychology at ELTE Eötvös Loránd University, Budapest, Hungary; 3 Faculty of Social Science, ELTE Eötvös Loránd University, Budapest, Hungary; 4 International Gaming Research Unit, Psychology Department, Nottingham Trent University, Nottingham, United Kingdom; 5 Centre of Excellence in Responsible Gaming at the University of Gibraltar, Gibraltar, Gibraltar; Universiti Pertahanan Nasional Malaysia, MALAYSIA

## Abstract

**Background:**

Work-related stress is significantly higher among healthcare workers (HCWs) than in the general population. Elevated occupational stress has been linked to burnout syndrome and depression. Moreover, medical professionals working during infectious disease outbreaks are at especially high risk for these problems. The aim of the present study was to examine the mental health status of HCWs and possible predictors of mental health status related to the COVID-19 outbreak utilizing a complex comprehensive model.

**Methods:**

In a countrywide cross-sectional survey among HCWs (N = 2087), work-related stress, COVID-19 -related objective work factors (displacement, frontline working) and subjective work factors (insecurity, unpredictability, workload), perceived stress, work-related stress, burnout and depression were assessed between the second and third wave of COVID-19 pandemic in Hungary.

**Results:**

COVID-19-related objective factors did not predict directly stress, burnout, and depression, whereas feelings of insecurity and unpredictability in relation to the COVID-19 situation at work had a significant medium-sized total effect (also considering the indirect effect via stress) on burnout and depression.

**Conclusions:**

In order to prevent subsequent mental health problems during crisis situations, such as the COVID-19 pandemic, healthcare management should create a more predictable work environment and a safer work experience for healthcare workers and provide mental health support.

## Introduction

Work-related stress has been found to be significantly higher among healthcare workers (HCWs) than among the general population, which also impacts negatively on their mental health [1–3]. The effects of elevated work stress on HCWs can vary widely and can lead to post-traumatic stress disorder (PTSD), depression, substance abuse, sleep disorders, and even suicide [4–6]. Elevated occupational stress has also been linked to burnout syndrome, and to deterioration in work performance [4, 7]. A longitudinal study, describing the relationship between burnout and work stress among UK physicians found circular causality: stress makes physicians more emotionally exhausted, and emotional exhaustion causes more stress [8].

According to a recent systematic review, psychiatric morbidity among physicians ranged from 17% to 52% [4]. Depression is one of the major mental health issues among HCWs. Cross-sectional studies have shown that depression ranges from 36.4% to 61.7% among HCWs, and work-related stress has been proven to be related to the development of clinical depression [9, 10]. Although depression and burnout syndrome are considered as separate nosological entities, numerous studies have reported a significant relationship between the two [11]. It has also been reported that job dissatisfaction can lead to depression (through burnout) and that it plays a more significant role in the development of depression than increased workload [12]. It has also been found that emotional exhaustion (EE) assessed by the EE subscale of the Maslach Burnout Inventory (MBI) correlates the most with the degree of depression, and that higher level of EE increases the risk of psychiatric morbidity among HCWs [13].

The emotional and mental vulnerability of HCWs is even more important during pandemics. As in any epidemiological crisis situations, HCWs are particularly exposed to stress at work. Therefore, the novel coronavirus disease 2019 (COVID-19) outbreak is likely to have had a significant impact on the mental health of HCWs. Among HCWs, the probability of infection is extremely high, and many professionals are forced to work in a changed work environment with an increased workload [14].

Previous research has suggested that medical professionals working during infectious disease outbreaks are at high risk for PTSD, depression, and burnout syndrome [15]. Earlier studies investigated the background factors of late onset clinical depression that occurred due to the stressful events of the SARS (severe acute respiratory syndrome) pandemic in 2003. Liu et al. [16] suggest that in addition to objective factors (e.g., sociodemographic factors and age, work exposure, pre-outbreak traumatic experience, quarantining), some perceived SARS-related factors (e.g., perceived risk, altruistic acceptance of risk) predicted late onset depression up to three years after the pandemic among medical professionals. High risk for PTSD has been also found among HCWs performing frontline tasks during the MERS (Middle East respiratory syndrome) outbreak in 2015 [17]. Several other occupational factors have been identified underlying psychological outcomes among HCWs during outbreaks of emerging infectious diseases. For instance, social support, perceptions of safety or risk, and the subjective impact on the individual’s life appear to play an important role in the development of mental health problems among HCWs [15]. Continuous psychiatric help for frontline workers during infectious disease outbreaks are recommended by mental health professionals to prevent PTSD and depression [17].

An umbrella review concerning the mental health impacts of the COVID-19 pandemic among HCWs reported a 24.9% prevalence of anxiety disorders and 24.8% prevalence of depression [18]. Another study reported that professionals working in direct contact with potential COVID-19 infected patients had high scores on Maslach Burnout Inventory (MBI) and on Perceived Stress Scale (PSS) than those who were not working on the frontline [19]. In contrast, a study performed in China reported lower levels of burnout syndrome among frontline HCWs compared to physicians not working in direct contact with COVID-19 infected patients [20]. One potential explanation for the lower level of burnout might be the greater sense of control and more accurate information concerning COVID-19 patient management [19].

Several studies have also examined the differences in mental health impact of viral outbreaks between nurses and physicians. Both previous studies carried out during the 2003 SARS outbreak, and the more recent ones during the COVID-19 pandemic have reported poorer mental health outcomes among nurses compared to other medical professional staff [21–23]. A Spanish study examined the relationship between burnout, depression, and perceived stress among HCWs and found similar stress levels, but significantly higher scores on the compassion fatigue and burnout subscales of the Professional Quality of Life Scale among nurses compared to physicians [19]. However, other studies have reported increased stress and depression levels among physicians compared to nurses [19, 24]. One possible explanation of these findings could be that physicians–due to difficult decisions–experience “moral injury” during the treatment of COVID-19 infected patients [25]. Moral injury is defined as a distress that occurs if decisions have to be made which violate professional ethical codes [25] and has been associated with PTSD and depression [26]. A good example of moral injury is that during the COVID-19 epidemic where physicians have to choose which patient to provide a higher level of medical care due to a lack of intensive care capacity [25].

It is important to highlight the predictive factors of high anxiety and depression levels among HCWs who feel less secure during their frontline work [27]. Many studies have examined the effects of inadequate isolation precautions, or shortage of personal protective equipment on stress and depression levels, but causal relationships have not been demonstrated by these studies [18]. While a relatively large number of descriptive studies have examined the mental health effects of COVID-19 outbreak among HCWs, systematic reviews have highlighted that very few have focused on the interrelationship between the background effects, burnout, depression, and anxiety in one model [18, 28].

The acute care of COVID-19 patients has been a significant burden on healthcare in Hungary. According to the Hungarian governmental webpage (koronavirus.gov.hu), the average number of new COVID-19 infections per million population during the study period was 257 (SD = 146). During the same period, the average number of hospital admissions per million people was 449 (SD = 83), and the average number of patients on ventilators per million people was 38 (SD = 11). The latest available official statistical data shows the number of acute hospital beds was 4271 per million inhabitants [29], which means more than 10% extra load for hospitals treating acute patients.

**Fig 1:**
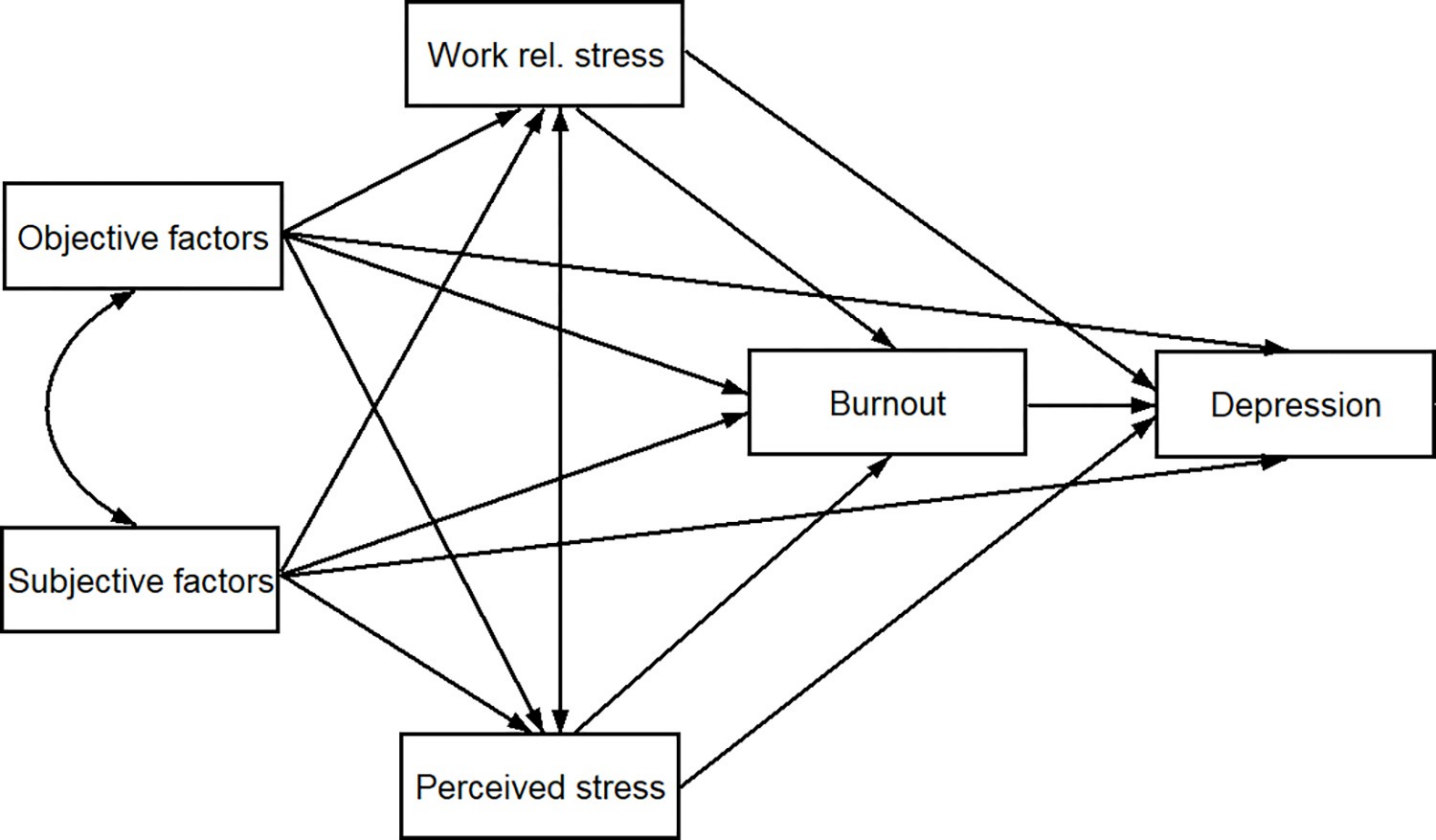
Hypothetical model of COVID-19 related factors’ possible effects on mental health.

Based on the aforementioned considerations, the aim of the present study was to examine the mental health status (including depression and burnout syndrome) of HCWs and its possible predictors related to the COVID-19 outbreak. Compared to previous studies, the present one examined the relationship between mental health indicators and the possible predictive factors in a complex model (Fig 1). The goal was to examine to what extent stress (directly and indirectly) explained depressive symptoms. Moreover, the proposed model aimed to (i) differentiate between the objective (e.g., displacement, frontline work) and subjective (e.g., uncertainty, unpredictability) COVID-19-related possible stress factors, as well as (ii) determine the differences between physician HCWs and non-physician HCWs.

## Methods

### Participants

Members of the Hungarian Medical Chamber (HMC) and Hungarian Chamber of Health Professionals (HCHP) were sent the link to the survey. A total of 3321 individuals started the survey during the data collection period between January 14 and March 5, 2021, between the second and third wave of COVID-19 pandemic in Hungary. The data of participants were included if they completed at least the 90% of the survey (N = 2260). Sixty-four respondents were excluded after anomaly detection in items of the psychometric scales using the SPSS ‘DETECTANOMLY’ function. A further 109 respondents who reported that they were not physicians or HCWs were also excluded from the final sample. The final sample, following omissions based on the aforementioned criteria, comprised 2087 individuals (85.82% female; 13.46% male; 0.72% not declaring their sex), with ages ranging from 21 to 87 years (M = 45.58 years, SD = 12.57). All participants were actively working as HCWs and none of the participants were retired. One-third were physician HCWs (33.78%) and 66.22% were non-physician HCWs; 38.06% worked in direct COVID-19 healthcare departments (either at COVID-19 or emergency departments of hospitals, or ambulance units), and 61.94% worked in non-COVID-19-related departments. Approximately one in six participants (16.72%) were relocated during the pandemic, while the remainder were not (83.28%).

### Procedure

APA ethical standards were followed in the conduct of the study, and the study was approved by the ELTE Eötvös Loránd University Research Ethics Committee. An online survey was developed using *Qualtrics*. The survey was pretested and adjusted in a pilot with HCWs. A link to the survey was sent to members of the HMC and HCHP accompanied by a short description about the study. All participants gave their informed and voluntary consent for participation and could withdraw at any time. Participants were given feedback based on evaluation of the Beck Depression Inventory. Psychological aid was offered for all participants, provided by a cooperating treatment service provider.

### Measures

#### COVID-19-related background factors

COVID-19-related background factors were assessed using self-developed items. Objective background factors were assessed by two items. One item assessed whether the respondent was working on the frontline (COVID-19 department irrespective of whether it was an ICU or non-ICU emergency department) or not. The other item assessed whether the HCW was relocated (i.e., had to work in a different department than usual) during the pandemic or not. Subjective background factors were assessed using three items. Each item (based on evaluation of a statement concerning changes in work-related factors compared to the time before the pandemic) is scored on a seven-item scale from 1 (*not agree at all*) to 7 (*fully agree*). The first item assessed changes in workload (*“The amount of work I do has increased”*), the second item assessed work-related insecurity (*“I feel less secure in my work”*), and the third item assessed work-related unpredictability (*“My job schedule is more unpredictable”*).

#### Mental health status related factors

Perceived stress was assessed using the four-item version of Perceived Stress Scale (PSS) [30] (Hungarian version [31]) comprising two positive and two negative items. Each item (e.g., *“In the last month*, *how often have you felt that you were unable to control the important things in your life*?*”*) is scored on five-point scale from 0 (never) to 4 (very often), with positive items reverse scored (scale range: 0–16, Cronbach alpha = 0.80).

Work-related stress was assessed using the Secondary Traumatic Stress element of Compassion Fatigue subscale of Professional Quality of Life Scale (5^th^ version) (PQL STS), developed as part of tool assessing quality of life among caregiving professionals [32] (Hungarian version [33]). The ProQL STS comprises 10 Likert type items. Each item (e.g., ‘I am preoccupied with more than one person I help.’) is scored on five-point scale from 1 (never) to 5 (very often) (scale range: 10–50, Cronbach alpha = 0.87).

Emotional exhaustion factor of burnout was assessed using the Emotional Exhaustion subscale of Maslach Burnout Inventory for Human Service Survey (MBI EE) [34] (Hungarian version [35]). The original version of MBI EE subscale comprised nine items assessing the frequency of specific work-related feelings. Each item (e.g., *“Feel emotionally drained from work”*) is scored on a seven-point scale from 0 (*never*) to 6 (*every day*). During the evaluation of the Hungarian version, Item 14 did not fit in the MBI EE subscale, therefore it was omitted (scale range: 0–48, Cronbach alpha = 0.94).

Depression was assessed using the shortened Hungarian version of the Beck Depression Inventory (BDI) [36, 37], developed by Kopp et al. [38]. The scale contains nine items related to symptoms of depression. Each item (e.g., ‘I’m too tired to do anything’) is scored on a four-point scale from 0 (not typical at all) to s (completely typical) (scale range: 0–27, Cronbach alpha = 0.86).

### Statistical analysis

Descriptive statistics were calculated for all variables of the study (i.e., means and standard deviations). Student *t*-tests were used for examining the difference between means of physician HCWs and non-physician HCWs. All variables were considered to be nearly normally distributed if skewness and kurtosis were in the range of +/-2 [39]. Skewness and kurtosis remained between -1.76 and +1.79 for all investigated variables in the present study. Cronbach’s α reliability estimation was conducted for psychometric scales. Correlation analysis was conducted by computing Pearson’s correlation coefficients with two-tailed significance tests. A *p* < .05 significance level was used for all statistical tests. SPSS v.23 was used for the descriptive statistics, reliability analysis, and correlation analysis.

The hypotheses were tested by structural equation modelling. The estimation method was selected according to normality check of the participating variables. All paths were removed from the model that were not significant (*p*>0.05). The goodness of fit of the model was tested by likelihood ratio tests (model versus baseline, model versus saturated), root mean square error approximation (RMSEA), comparative fit index (CFI), Tucker-Lewis index (TLI), goodness of fit index (GFI). A model is considered to fit well if RMSEA<0.06, TLI, CFI >0.95 and GFI>0.90 [40]. Unstandardized and standardized coefficients as well as total effect (calculated from direct and indirect effect) and equation-level goodness of fit (R^2^) were calculated in connection with the final model. An unconstrained and constrained multigroup structural equation model was used in order to test structural invariance across physician and non- physician HCWs. Stata 14 was used for all calculations for the SEM models.

## Results

### Descriptive statistics and preliminary analyses

Table 1 provides descriptive statistics of the study variables. There was no significant difference between physician HCWs and non-physician HCWs in relation to COVID-19-related objective factors. In relation to COVID-19-related subjective factors, HCWs perceived an increase the in level of insecurity (M = 4.89), with no significant difference between physician HCWs and non-physician HCWs, whereas non-physician HCWs perceived significantly higher increase in workload (M = 5.31) and unpredictability (M = 4.72) than physicians (M = 4.62; M = 4.50 respectively). There were no significant differences between physician HCWs and non-physician HCWs in relation to stress, work-related stress, and burnout, but non-physician HCWs reported significantly higher depression scores than physician HCWs.

**Table 1:** Comparative descriptive statistics of the study variables between physicians and non-physician HCWs.

	Physicians			Non-physician healthcare workers			Total				
Variables	n	M	SD	n	M	SD	n	M	SD	Skewness	Kurtosis
1. Frontline	684	.38_a_	.49	1,268	.38_a_	.49	1,952	.38	.49	0.49	-1.76
2. Displaced	705	.15_a_	.35	1,382	.18_a_	.38	2,087	.17	.37	1.79	1.19
3. Insecurity	703	4.98_a_	1.95	1,369	4.85_a_	1.98	2,072	4.89	1.97	-0.57	-0.88
4. Unpredictability	702	4.50_a_	2.24	1,371	4.72_b_	2.21	2,073	4.64	2.22	-0.42	-1.29
5. Workload	700	4.62_a_	2.18	1,376	5.31_b_	1.95	2,076	5.08	2.05	-0.71	-0.77
6. Work-related stress (ProQoL STS)	675	23.59_a_	7.40	1,285	24.20_a_	7.57	1,960	23.99	7.52	0.45	-0.25
7. Perceived stress (PSS)	702	7.04_a_	3.31	1,369	7.32_a_	3.08	2,071	7.22	3.16	-0.09	-0.46
8. Burnout (MBI EE)	701	23.80_a_	13.26	1,372	24.91_a_	12.93	2,073	24.54	13.05	-0.01	-1.01
9. Depression (BDI)	705	6.64_a_	5.20	1,381	7.32_b_	5.24	2,086	7.09	5.24	0.58	-0.44

Note. Means for physician HCWs and non-physician HCWs in the same row not sharing the same subscript are significantly different at *p* < .05 in the two-sided test of equality for column means. Cells with no subscript are not included in the test. Tests assume equal variances. ProQol STS: Professional Quality of Life Scale, Compassion Fatigue subscale; PSS: Perceived Stress Scale; MBI EE: Maslach Burnout Inventory for Human Service Survey; BDI: Beck Depression Inventory

Table 2 provides Pearson correlations between all study variables. According to power analysis, a 0.07 correlation in the population can be detected with type II error level of .05. All correlations were found to be significant except for correlations between age and increased insecurity, sex and (i) working on the frontline, (ii) working in displaced departments, and (iii) increased unpredictability. All correlations between perceived stress, work-related stress, burnout, and depression were high and significant (between .55 and .72). Correlations between COVID-19-related subjective factors and mental health indicators were moderately significant (between .22 and .38) except for the weak correlation between increased workload and work-related stress (.19). Correlations between COVID-19-related objective factors and mental health indicators were weak (between .08 and .17). The two COVID-19-related objective factors were strongly correlated (.44) while the correlation between subjective factors were of moderate strength (between .23 and .37).

**Table 2:** Pearson correlations for study variables.

Variables	n	1	2	3	4	5	6	7	8	9	10	11
1. Age	2,006	-										
2. Sex	2,072	-.07**	-									
3. Frontline ^a^	1,952	-.34**	-.09**	-								
4. Displaced ^b^	2,087	-.19**	.07**	.44**	-							
5. Insecurity	2,072	-.02	.05*	.00	-.01	-						
6. Unpredictability	2,073	-.18**	.03	.22**	.20**	.31**	-					
7. Workload	2,076	-.11**	.14**	.21**	.14**	.23**	.37**	-				
8. Work-related stress (ProQoL STS)	1,960	-.12**	.14**	.08**	.09**	.29**	.23**	.19**	-			
9. Perceived stress (PSS)	2,071	-.25**	.13**	.12**	.10**	.29**	.31**	.23**	.55**	-		
10. Burnout (MBI EE)	2,073	-.27**	.11**	.16**	.13**	.34**	.38**	.32**	.58**	.66**	-	
11. Depression (BDI)	2,086	-.27**	.10**	.17**	.14**	.28**	.33**	.22**	.59**	.72**	.72**	-

Note.

^a^ 0 = not working in the frontline 1 = working in the frontline

^b^ 0 = were not displaced 1 = were displaced

* *p* < .05^,^

**p < .01

ProQol STS: Professional Quality of Life Scale, Compassion Fatigue subscale; PSS: Perceived Stress Scale; MBI EE: Maslach Burnout Inventory for Human Service Survey; BDI: Beck Depression Inventory

### Structural equation model

Maximum likelihood model with missing values was used for fitting the model as all criteria were met. All fit statistics for final model summarized in Table 3 indicated close fit. The unconstrained multigroup model for physicians and non-physician HCWs also showed a good fit. The constrained multigroup model with equal structural coefficients and error variance showed close fit for all fit statistics except for the χ^2^ test. However, χ^2^/df was 2.1 which is near to or under recommended thresholds [41, 42].

**Table 3:** Fit statistics for the structural equation models.

Model	*χ* ^2^	*df*	*P*	CFI	TLI	GFI	RMSEA	Model AIC
Final full model	18.45	11	.072	.99	.99	.99	.018 (90% CI: .000, .031)	96516.8
Unconstrained multigroup model	33.10	22	.060	.99	.99	.99	.022 (90% CI: .000, .037)	94786.2
Multigroup model assuming structural invariance	111.20	53	.000	.99	.99	.98	.032 (90% CI: .024, .041)	94782.0

**Fig 2:**
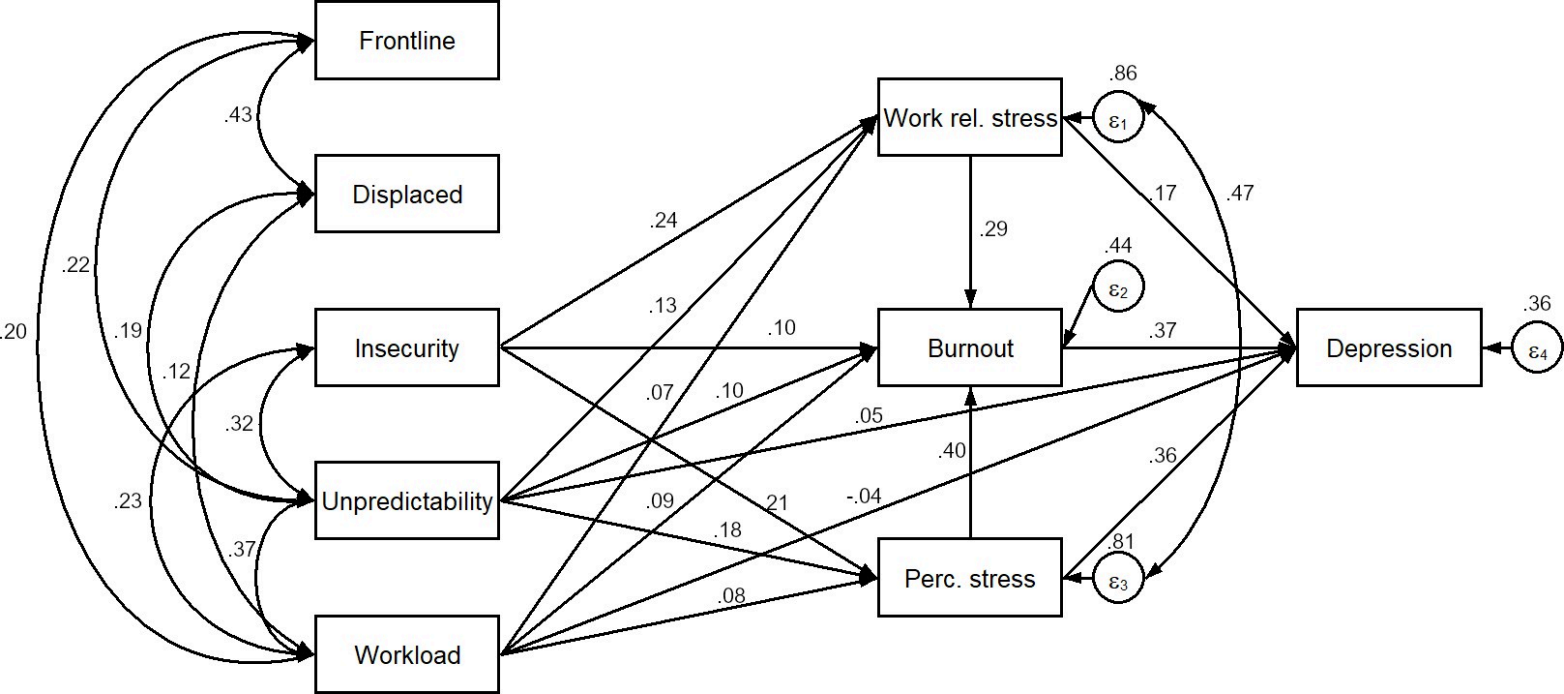
Relationship between COVID-19 related factors and mental health (final SEM model). Note. Control variables (age, sex) and non-significant correlations are not presented. Figure shows standardized coefficients, correlations and variances of error terms. ε_1–4_ error terms of the four equations.

In the final SEM model (Fig 2), there were no significant relationships between COVID-19-related objective factors and any of mental health indicators. However, COVID-19-related objective factors had significant but weak to medium correlations with COVID-19-related subjective factors. One of the COVID-19-related subjective factors (i.e., increased insecurity) had a medium positive direct effect (0.21 to 0.24) on perceived stress and work-related stress, a weak direct effect on burnout, and non-significant effect on depression. Another COVID-19-related subjective factor (i.e., increased unpredictability) also had a weak positive direct effect (0.05 to 0.18) on perceived stress, work-related stress, burnout, and depression. The third COVID-19-related subjective factor (i.e., increased workload) had a very weak positive direct effect (0.07 to 0.09) on perceived stress, work-related stress, and burnout, and a very weak adverse effect on depression (-0.04). Considering both direct and indirect effects (Table 4), effects of all COVID-19-related subjective factors were positive on both burnout and depression. Two factors (i.e., increased insecurity and unpredictability) had a medium effect on burnout and depression, while increased workload had a weak effect on burnout and depression.

**Table 4:** Total effects of COVID-19-related subjective factors, stress, and control variables on burnout and depression.

		Std. Coeff.	z	*p*>|z|
Burnout	Perceived stress	.40	21.70	.000
	Work-related stress	.29	15.80	.000
	Insecurity	.25	12.35	.000
	Unpredictability	.21	10.04	.000
	Workload	.14	7.02	.000
	*Age*	-.20	-10.16	.000
	*Sex*	.07	5.44	.000
Depression	Burnout	.37	18.87	.000
	Perceived stress	.51	26.03	.000
	Work-related stress	.28	14.99	.000
	Insecurity	.21	12.85	.000
	Unpredictability	.21	9.71	.000
	Workload	.06	2.65	.008
	*Age*	-.19	-9.56	.000
	*Sex*	.07	5.38	.000

Perceived stress had a strong total effect on burnout and depression, while work-related stress had a medium total effect on burnout and depression. The explained variance was 64% for depression, 56% for burnout, 14% for work-related stress, and 19% for perceived stress according to the model. The coefficient of determination for the total model was 0.31.

## Discussion

The hypothesized model concerning the effect of COVID-19-related objective and subjective factors on stress, burnout, and depression was partially supported by the SEM model. According to the results, both perceived stress and work-related stress increased burnout confirming results of previous research (e.g., [4, 7]). It was also found that general perceived stress and work-related stress were both directly and indirectly related to depression as previously suggested by Tokuda [12]. The effect of COVID-19-related factors as stressors were also confirmed.

However, in a major difference to the hypothesized model, no direct effect of COVID-19-related objective factors (e.g., working on the frontline or having to work in another department during the pandemic) to stress, burnout, or depression was found. Based on the results, it appears that the difficulties caused by the pandemic only had a mediated effect. Because stressors exert their effect through subjective perception only, it underpins the importance of mental health support among HCWs in times of extreme crisis situations as suggested by Lee et al. [17]. Consistent with the present study’s research findings, numerous studies have highlighted the negative impact of feelings of uncertainty on mental health among frontline HCWs (e.g., [43, 44]). These findings underline that specific intervention programs for HCWs are important because the symptoms and additional consequences of depression and burnout often appear years later as emphasized earlier in the paper [17].

Comparing physician HCWs and non-physician HCWs, no differences were found in the mechanisms of how COVID-19-related factors may cause stress, burnout, and depression. Therefore, in the model proposed, similar background factors may play a role in both HCW sub-populations. However, the study found that non-physician HCWs perceived a higher increase in work insecurity and in workload (COVID-19-related subjective factors) than physician HCWs and they also had higher mean depression scores compared to physician HCWs, although there were no significant differences between the two groups in the level of stress or burnout. These seemingly controversial results can be understood based on the model explained above. The differences in COVID-19-related subjective factors through direct and indirect paths only yielded a significant difference in the level of depression while the difference in the level of the partial mediator stress and burnout were not significant. Other researches comparing stress, burnout and depression among physician HCWs and non-physician HCWs were controversial. While Ruiz‐Fernández [19] found similar stress level in the two groups but increased compassion fatigue and burnout among non-physician HCWs, Salari et al. [24] reported increased stress and depression levels among physician HCWs.

The present study’s sample comprised high proportion of female HCWs. There could be additional care burdens in the case of female HCWs due to the impact of the COVID-19 pandemic within their own families [45]. However, the findings indicated only a minimal effect of sex in the proposed model regarding HCWs. The total effect of sex was significant but very small on both burnout and depression (Table 4). The same also holds true for the relationship between sex and work-related subjective factors (Table 2).

The study has a number of limitations that should be considered when interpreting the findings. While the sample size was large and all members of the HMC and HCHP had the opportunity to participate in the survey, the participants were recruited using convenience sampling. Moreover, the data collected were self-report (which is subject to well established methods biases) and cross-sectional in nature (and therefore causal interpretations should be noted with caution). The findings were supported by both theoretical literature and previous research which decrease the possibility of misinterpretation of causality. The survey was administered online that which limited the length of the questionnaire. This limitation meant other important factors were not assessed such as resilience, social support, and more detailed questions concerning working conditions. Assessing and analyzing these factors in further research with a face-to-face study would help the in-depth understanding of the relationships examined in the present study. In-depth understanding of the psychological mechanisms underlying the findings could also be supported by further semi-structured interviews in future research.

Based on the results reported here, in order to prevent subsequent mental health problems during crisis situations, such as the COVID-19 pandemic, it is extremely important to provide effective and accurate information to HCWs on safe patient care, to help create a predictable work environment in order to increase sense of security. The importance of greater sense of control and more accurate information concerning COVID-19 patient management was also emphasized by Ruiz‐Fernández et al. [19]. The results also indicate that non-physician HCWs might even be more vulnerable to COVID-19-related crisis than physicians. This may be because there is less support and supervision given to non-physician HCWs by co-workers [2]. The quality of relationships with co-workers as a protective factor against burnout was also emphasized by Poncet [46]. As a possible solution, confidential intervention and online mental health support [5] could be offered specifically to non-physician HCWs to help them to cope with increased work-related stress, especially because targeted mental health prevention has extreme importance.
